# Global research trends in the COVID-19 and digestive disease: A review of visualization and bibliometric study

**DOI:** 10.1097/MD.0000000000032705

**Published:** 2023-01-20

**Authors:** Peiling Gan, Shu Huang, Xiao Pan, Huifang Xia, Xinyi Zeng, Wensen Ren, Lei Shi, Muhan Lü, Xian Zhou, Xiaowei Tang

**Affiliations:** a Department of Gastroenterology, The Affiliated Hospital of Southwest Medical University, Luzhou, China; b Nuclear Medicine and Molecular Imaging Key Laboratory of Sichuan Province, Luzhou, China; c Department of Gastroenterology, The People’s Hospital of Lianshui, Huaian, China.

**Keywords:** COVID-19, digestive disease, visualization, VOSviewer, CiteSpace

## Abstract

The rapid spread of coronavirus disease 2019 (COVID-19) has attracted worldwide attention. There were also reported gastrointestinal symptoms in patients with COVID-19. This work aims to analyze the global research trends in COVID-19 and digestive disease.

**Methods:**

The related papers on COVID-19 and digestive disease were identified with Pubmed and web of science core collection on September 3, 2021. Bibliometric visualization was conducted through VOSviewer and CiteSpace.

**Results:**

The analytic research was based on original articles and reviews. There were 997 articles found, with citations ranging from 0 to 878. These articles were distributed among 86 countries and 355 journals. The USA mainly contributed (288 articles), where 3 of the top 10 institutions were located. Followed by China (215 articles) and Italy (160 articles). The highest level of scientific collaboration has been formed between the USA to China. The *World Journal of Gastroenterology* (39 papers) published the most significant number of articles. Concerning the research topic, the colon/small bowel had the largest number of articles, followed by the liver and pancreaticobiliary. “Liver injury,” “inflammatory bowel disease,” “management,” and “endoscopy” were the hotspot keywords. The largest cluster of liver transplantation had offered hints regarding research frontiers.

**Conclusion:**

The analytic results showed that the liver, especially liver transplantation, and inflammatory bowel disease were the 2 most influential research topics in COVID-19 and digestive disease.

## 1. Introduction

A cluster of unexplained pneumonia cases caused by a novel coronavirus, severe acute respiratory syndrome coronavirus 2 (SARS-CoV-2), was initially reported in early December 2019 in Wuhan, Hubei Province, China.^[1]^ The virus swiftly spread throughout the rest of China and then internationally over a relatively short period. The world health organization named this outbreak as coronavirus disease 2019 (COVID-19) and characterized it as a pandemic on March 11, 2020.^[2]^ The rapid spread of COVID-19 has attracted worldwide attention. As of September 24, 2021, the cumulative numbers of confirmed cases and deaths globally were reported to be nearly 229 million and over 4.7 million, respectively, by world health organization.^[3]^ The SARS-CoV-2 is still evolving and mutating. Song C et al also reviewed the origins, pathogenic mechanisms, transmission characteristics, detection and diagnosis, evolution, and variation of SARS-CoV-2,^[4]^ indicating that the risk of its potential rapid evolution should not be ignored. In addition, patients with COVID-19 may begin excreting or secreting the virus before symptoms appear, meaning that the emergence of asymptomatic infections poses a higher demand for pandemic control.^[4]^

Once an individual is infected with COVID-19, the most common symptoms are fever, cough, myalgia, or fatigue,^[5]^ which are similar to the symptoms of the severe acute respiratory syndrome (SARS) reported in 2003 and the Middle East respiratory syndrome reported in 2012.^[6]^ Gastrointestinal symptoms, such as diarrhea, were also reported in patients with COVID-19. A study revealed that the pooled prevalence of all gastrointestinal symptoms, including anorexia, nausea/vomiting, diarrhea, and abdominal pain/discomfort, was 17.6%.^[7]^ Some comorbidities of the digestive system can also significantly affect the prognosis of patients with COVID-19.^[8]^

In this study, we aimed to evaluate the global research trends in COVID-19 and digestive disease and present new inspirations for scientific research using this bibliometric analysis.

## 2. Methods

### 2.1. Data collection and search strategy

**Figure 1.**
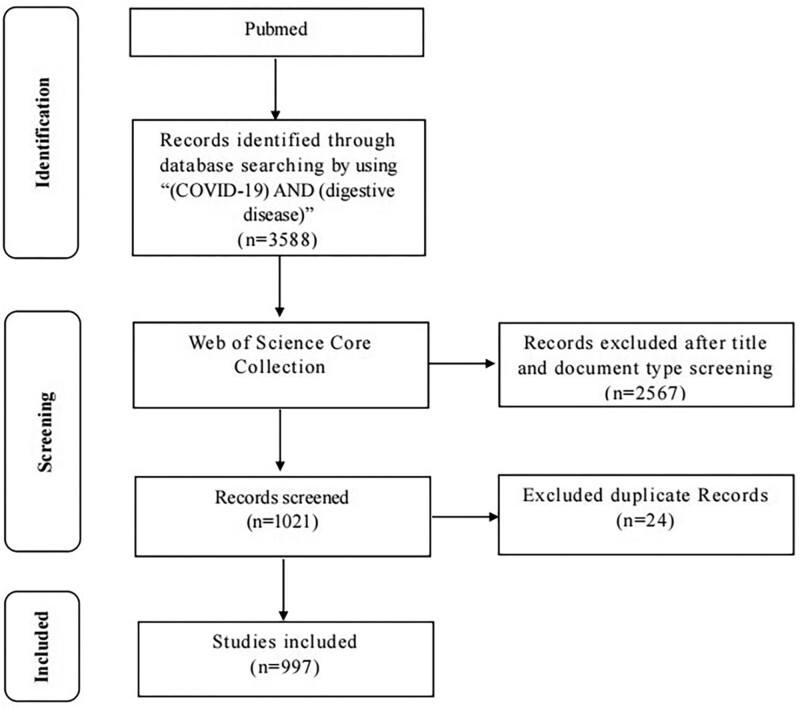
A flowchart representing search strategies from the Web of Science Core Collection (WoSCC) database. WoSCC = web of science core collection.

The bibliometric analysis was carried out on September 3, 2021. Pubmed was used to search all relevant papers. The following was the search strategy: All fields = (COVID-19) AND (digestive disease). The web of science core collection database was used to verify all eligible papers, limit the types of articles and identify the number of citations. Only articles or review articles were included. After excluding unqualified and duplicate records, we ended up with 997 records. Then we sorted all the retrieved records in descending order according to the number of citations for further analysis. A flowchart representing retrieval strategies is shown in Figure 1.

### 2.2. Analysis tool

VOSviewer and CiteSpace were chosen. Some parameters were collected and analyzed, such as article counts, number of citations, citations per article, and the impact factor. Productivity was measured by the published article numbers, and used to identify productive individuals or groups. The impact factor was obtained referring to journal citation reports 2020.

The network visualization maps were constructed using VOSviewer to examine the cooperative relationships. Co-authorship analysis identified research output. Keywords with the highest citation numbers were selected to demonstrate research hotpots.

CiteSpace V adopts a time-slicing technique to create a timeline of network models and integrates these individual networks to produce an overview network for the systematic analysis of the relevant publications. We conducted a co-citation analysis of the references and clusters, and a timeline view of co-cited references was built. These can identify whether relevant scholars have paid extensive attention to these areas in a specific period.

## 3. Results

### 3.1. Publication output

**Figure 2.**
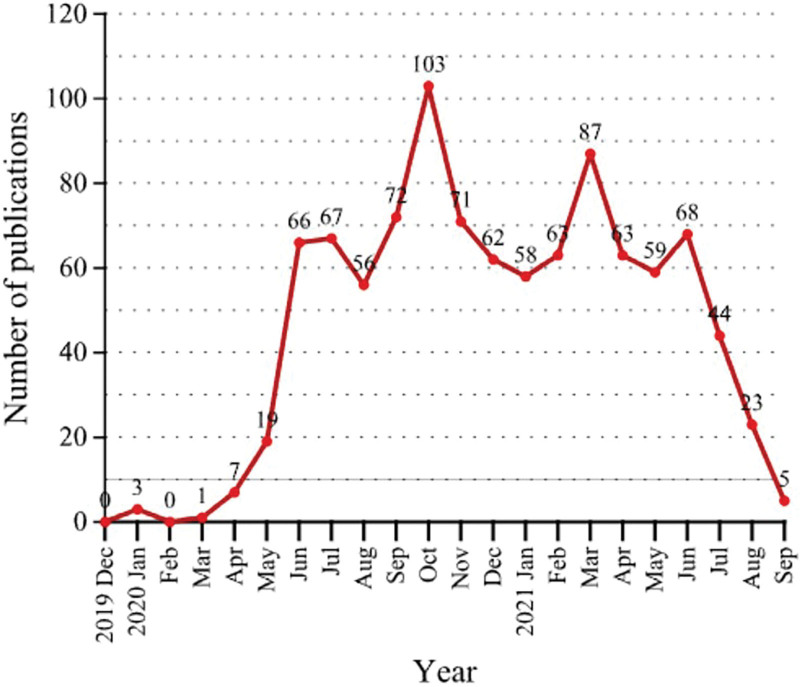
Trends of the worldwide publication output of COVID-19 and digestive disease. COVID-19 = coronavirus disease 2019.

A total of 997 articles fulfilled the criteria described above. Figure 2 shows the trends of the worldwide publication output of COVID-19 and digestive disease. Table 1 lists the top 10 cited articles in descending order. The 100 top-cited articles on COVID-19 and digestive disease are available in Table S1, Supplemental Digital Content, http://links.lww.com/MD/I343. All these top-10 articles were published in 2020. Among the ten most cited papers, there were 5 comprehensive narratives related to gastrointestinal symptoms,^[9–13]^ 2 related to the liver,^[14,15]^ 1 related to endoscopy,^[16]^ 1 related to Gut Microbiota,^[17]^ and 1 related to SARS-CoV-2 infection of human small intestinal enterocytes.^[18]^ The article “Hepatic involvement in COVID-19 patients: Pathology, pathogenesis, and clinical implications” by Li Y et al^[14]^ had the most total citations (878 times).

**Table 1:** The top 10 cited articles of WoSCC bibliometrics.

Rank	First author	Journal	Title	Number of citations (WoSCC)	Type of articles
1	Li Y	J Med Virol. 2020;92(9):1491–1494.	Hepatic involvement in COVID-19 patients: Pathology, pathogenesis, and clinical implications	878	Review
2	Jin X	Gut. 2020;69(6):1002–1009.	Epidemiological, clinical and virological characteristics of 74 cases of coronavirus-infected disease 2019 (COVID-19) with gastrointestinal symptoms	491	Retrospective Study
3	Pan L	Am J Gastroenterol. 2020;115(5):766–773.	Clinical Characteristics of COVID-19 Patients with Digestive Symptoms in Hubei, China: A Descriptive, Cross-Sectional, Multicenter Study	441	Multicenter Study
4	Tian Y	Aliment Pharmacol Ther. 2020;51(9):843–851.	Review article: gastrointestinal features in COVID-19 and the possibility of fecal transmission	315	Review
5	Lin L	Gut. 2020;69(6):997–1001.	Gastrointestinal symptoms of 95 cases with SARS-CoV-2 infection	307	Retrospective Study
6	Fan Z	Clin Gastroenterol Hepatol. 2020;18(7):1561–1566.	Clinical Features of COVID-19-Related Liver Functional Abnormality	291	Retrospective Study
7	Zang R	Sci Immunol. 2020;5(47):eabc3582.	TMPRSS2 and TMPRSS4 promote SARS-CoV-2 infection of human small intestinal enterocytes	278	Clinical Trial
8	Wong SH	J Gastroenterol Hepatol. 2020 May;35(5):744–748.	Covid-19 and the digestive system	267	Review
9	Repici A	Gastrointest Endosc. 2020;92(1):192–197.	Coronavirus (COVID-19) outbreak: what the department of endoscopy should know	244	Review
10	Zuo T	Gastroenterology. 2020;159(3):944–955.e8.	Alterations in Gut Microbiota of Patients With COVID-19 During Time of Hospitalization	229	Clinical Trial

COVID-19 = coronavirus disease 2019, SARS-CoV-2 = severe acute respiratory syndrome coronavirus 2, WoSCC = web of science core collection.

### 3.2. Distribution by countries, institutions, journals, and authors

**Figure 3.**
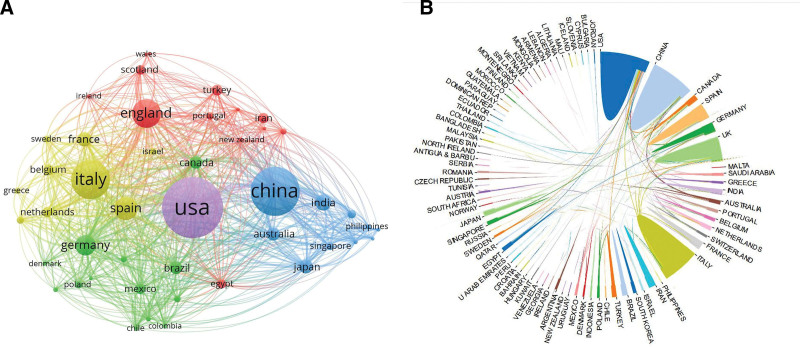
Scientific production and collaboration by countries. (A) Influential countries. (B) Visualization mapping of collaborating countries.

**Figure 4.**
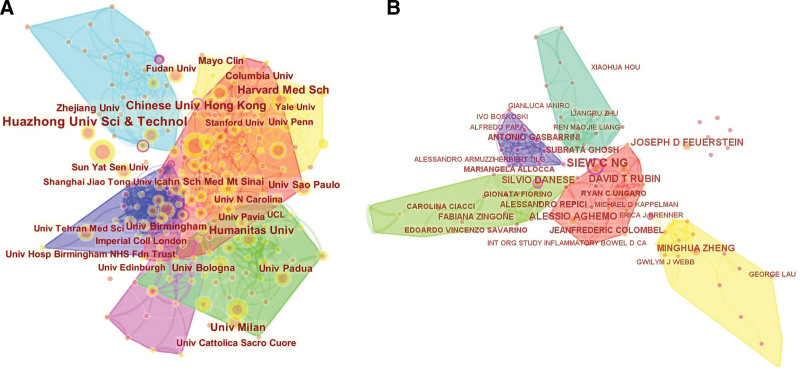
Scientific influence of COVID-19 and digestive disease research worldwide. (A) Influential institutions (B) Influential authors. COVID-19 = coronavirus disease 2019.

All of the papers came from 86 countries. Table 2 lists detailed information on the top 10 countries. The USA had the most publications (288 papers), followed by China (215 papers) and Italy (160 papers). Collaborations between countries are depicted in Figure 3. The different widths of the colored lines indicated the different scales of collaboration. The highest level of scientific collaboration has been formed between the USA to China. Figure 4 shows the most influential institutions and authors. Table 3 shows the most influential institutions and journals. The Huazhong University of Science and Technology (41 papers) ranked first, followed by The Chinese University of Hong Kong (32 papers) and the Harvard Medical School (25 papers). Among the top 10 institutions, 4 are located in China, 3 are in the USA, and 2 are in Italy. The most influential author, Siew C Ng, from the Chinese University of Hong Kong, had the most papers. The *World Journal of Gastroenterology* published the most papers (39 papers), followed by *Gut* (28 papers) and *Digestive and Liver Disease* (26 papers). *Gastroenterology* (68.50) and *Gut* (66.18) had the largest citation/article ratio.

**Table 2:** The top 10 most productive countries.

Rank	Country	Number of articles	Number of citations	Citations per article
1	USA	288	4691	16.29
2	China	215	7834	36.44
3	Italy	160	2503	15.64
4	England	106	1926	18.17
5	Spain	80	1332	16.65
6	Germany	67	1148	17.13
7	India	49	811	16.55
8	France	44	719	16.34
9	Japan	40	374	9.35
10	Australia	39	567	14.54

**Table 3:** The top 10 most productive institutions and journals.

Rank	Institutions	No. of articles	No. of citations	Citations per article	Country	Rank	Journal	No. of articles	Total citations	Citations per article	IF (2020)
1	Huazhong University of Science and Technology	41	1449	35.34	CHINA	1	World Journal of Gastroenterology	39	174	4.46	5.742
2	The Chinese University of Hong Kong	32	1487	46.47	CHINA	2	Gut	28	1853	66.18	23.059
3	Harvard Medical School	25	729	29.16	USA	3	Digestive and Liver Disease	26	195	7.50	4.088
4	Wuhan University	21	1533	73.00	CHINA	4	Gastroenterology	22	1507	68.50	22.682
5	Humanitas University	21	752	35.81	ITALY	5	American Journal of Gastroenterology	18	824	45.78	10.864
6	Columbia University	19	325	17.10	USA	6	Bmj Open	18	25	1.39	2.692
7	University of Milan	18	313	17.39	ITALY	7	Journal of Gastroenterology and Hepatology	17	429	25.24	4.029
8	Icahn School of Medicine at Mount Sinai	18	265	14.72	USA	8	Inflammatory Bowel Diseases	17	200	11.76	5.325
9	Zhejiang University	17	903	53.12	CHINA	9	Hepatology International	15	265	17.67	6.047
10	University of Birmingham	15	1060	70.67	ENGLAND	10	Journal of Crohns & Colitis	15	202	13.47	9.071

IF = impact factor.

### 3.3. Research topics and keywords

**Figure 5.**
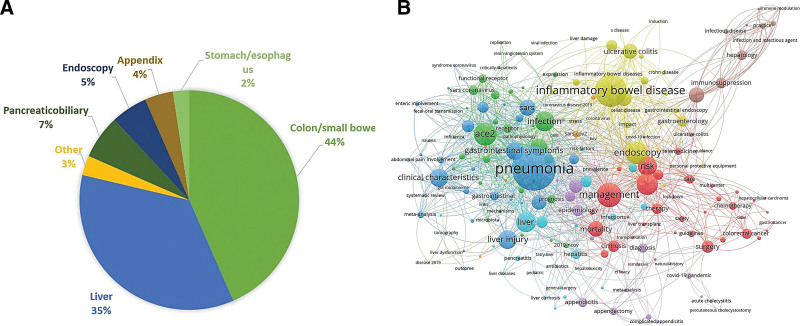
Research hotspots. (A) Hotspots of research topic. (B) Hotspots of keywords.

The research topics of these papers are shown in Figure 5A. The essential research topics were as follows: Colon/small bowel (436 papers, 44%), Liver (354 papers, 35%), Pancreaticobiliary (65 papers, 7%), and Endoscopy (54 papers, 5%). Figure 5B displays the most influential keywords. Among them, “liver injury,” “inflammatory bowel disease,” “management,” and “endoscopy” were particularly worthy of attention.

### 3.4. Analysis of references

**Figure 6.**
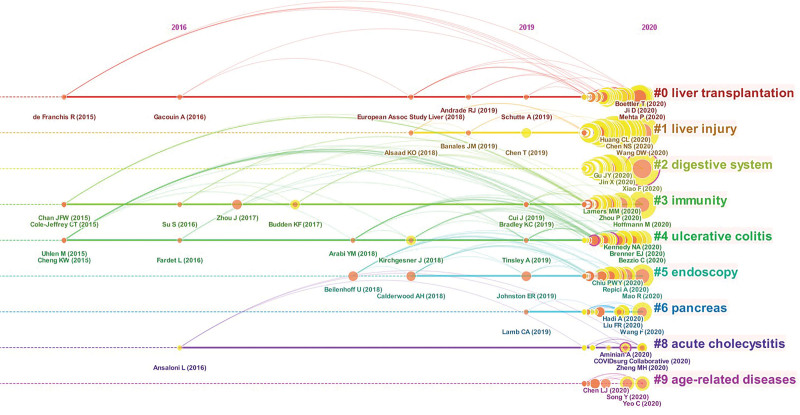
A timeline visualization of the largest clusters of COVID-19 and digestive disease. (From left to right, each cluster was presented. The publication time legend was displayed at the top. The clusters were arranged vertically and in declining order of size. The largest cluster was presented at the top). COVID-19 = coronavirus disease 2019.

In bibliometric research, reference analysis is an important indication. Clusters along with horizontal timelines were depicted in Figure 6, which was a timeline visualization in CiteSpace. The 3 most cited references in a given year were listed below each timeline. The label of the most cited reference was displayed at the bottom of the timeline. Cluster #0 was the largest cluster.^[19]^ As shown in the timeline overview, the largest cluster in this study was #0 liver transplantation, indicating a significant research interest. Followed by #1 liver injury, #2 digestive system, #3 immunity, #4 ulcerative colitis, #5 endoscopy, and #6 pancreas. The most cited references of each of these clusters are listed in Table 4.

**Table 4:** The most cited references of each cluster.

Cluster ID	Label	The most cited reference
0	Liver transplantation	Mehta P, et al COVID-19: consider cytokine storm syndromes and immunosuppression. *Lancet*. 2020;395:1033–4.
1	Liver injury	Wang D, et al Clinical characteristics of 138 hospitalized patients with 2019 novel coronavirus-infected pneumonia in Wuhan, China. *JAMA*. 2020;323:1061–9.
2	Digestive system	Xiao F, et al Evidence for gastrointestinal infection of SARS-CoV-2. *Gastroenterology*. 2020;158:1831–3.e3.
3	Immunity	Hoffmann M, et al SARS-CoV-2 cell entry depends on ACE2 and TMPRSS2 and is blocked by a clinically proven protease inhibitor. *Cell*. 2020;181:271–80.e8.
4	Ulcerative colitis	Bezzio C, et al Outcomes of COVID-19 in 79 patients with IBD in Italy: an IG-IBD study. *Gut*. 2020;69:1213–7.
5	Endoscopy	Mao R, et al Implications of COVID-19 for patients with preexisting digestive diseases [published correction appears in Lancet Gastroenterol Hepatol. 2020 Jul;5(7):e6]. *Lancet Gastroenterol Hepatol*. 2020;5:425–7.
6	Pancreas	Wang F, et al Pancreatic injury patterns in patients with coronavirus disease 19 pneumonia. *Gastroenterology*. 2020;159:367–70.

COVID-19 = coronavirus disease 2019, IBD = inflammatory bowel disease, SARS-CoV-2 = severe acute respiratory syndrome coronavirus 2.

## 4. Discussion

The impact of COVID-19 has been devastating worldwide due to the high pathogenicity and tendency to spread easily.^[20]^ National healthcare systems have been overwhelmed, leading to nationwide quarantines, stagnation of international travel, shortages of protective equipment, and global economic recession.^[21]^ Treatment of COVID-19 patients is a common challenge, and global cooperation is needed to overcome this pandemic. Some comorbidities can also significantly affect the prognosis of patients with COVID-19.^[8]^ There were also reported gastrointestinal symptoms in patients with COVID-19. Therefore, the present bibliometric analysis was conducted to explore the global research trends between digestive diseases and COVID-19.

The USA and China made a significant contribution to this area. Acute liver injury is of great concern to both China and the USA. Institutes or authors from the USA and China were involved in 1 and 9 of the top 10 most-cited articles and 43 and 41 of the top 100 most-cited articles, respectively. Universities are the most common research groups. Four institutions are located in China, 3 are in the USA, and 2 are in Italy. *Gastroenterology* focused on the characteristics of the digestive system involvement of COVID-19. In contrast, the *Gut* focused more on the liver and inflammatory bowel disease (IBD). Both journals exhibited the highest academic quality.

Of the 997 papers on COVID-19 and digestive disease, 35% focused on liver-related issues. Although lung injury is the primary clinical manifestation of COVID-19, laboratory testing has revealed substantial abnormalities in other organs, such as liver injuries.^[22–24]^ Fan Z et al reported that abnormal liver tests are common in COVID-19 patients, and SARS-CoV-2 may cause liver function damage (ranked 6th, cited 291 times).^[15]^ Zhang et al reported liver injury in 14% to 53% of COVID-19 patients, including 2% to 11% with underlying chronic liver disease.^[25]^ According to Li Y et al, liver damage was found in a substantial proportion of patients over the clinical course of COVID-19, particularly those with severe or critical diseases.^[14]^ The pathologic changes observed included a mild increase in sinusoidal lymphocytic infiltration, sinusoidal dilatation, steatosis, and multifocal hepatic necrosis. They believe 2 likely underlying mechanisms are potential hepatotoxicity from treatment medicines and direct viral-induced cellular damage (ranked top, cited 878 times).^[14]^ Furthermore, preexisting chronic liver disease may worsen during COVID-19, and the hyperinflammatory reactions associated with COVID-19 may also lead to liver damage.

It is crucial to highlight that there is currently no widespread agreement on therapeutic options for hepatic damage in COVID-19 patients. The pivotal point is prevention, which includes assisting patients with underlying liver diseases to reduce their risks of infection and preventing liver damage by reducing the use of medicines with potential liver toxicity. Hence, the interaction of viral hepatitis and COVID-19 is particularly worthy of concern. A consensus statement discussed how COVID-19 might impact liver transplant providers and their patients and listed the clinical best practice advice, which has great reference value.^[26]^

A meta-analysis performed by Cheung et al demonstrated that the pooled incidences of anorexia, nausea/vomiting, diarrhea, and abdominal pain/discomfort were 26.8%, 10.2%, 12.5%, and 9.2%, respectively.^[7]^ Ye et al suggested that the mechanism underlying gastrointestinal symptoms associated with COVID-19 might involve damage to the intestinal mucosal barrier and subsequent increased production of inflammatory factors.^[27]^ Zang R et al conducted a study to determine whether SARS-CoV-2 infects human intestinal epithelial cells (ranked 7th, cited 278 times).^[18]^ They found that the intestine is a potential site of SARS-CoV-2 replication, where the virus enters the host cell more easily, contributing to local and systemic illness and overall disease progression.

Colon/small bowel disease, especially IBD, warrants significant attention. Pan Y et al tested stool samples from 17 confirmed patients using RT-PCR and found that 9 of them were positive.^[28]^ Liu Y. et al revealed the aerodynamic characteristics of the SARS-CoV-2 aerosol transmission route and proposed the viral aerosol propagation model.^[29]^ This presents a unique challenge for endoscopy examination, especially for managing IBD patients. More notably, patients with IBD are more likely to become infected, especially if they are on immunosuppressants or biologics.^[30]^ In IBD patients, increasing age, comorbidities, and use of corticosteroids are related to severe COVID-19. However, according to Bezzio C et al, IBD patients are not at a higher risk of COVID-19 infection than the general population. Furthermore, combined therapy with biologics and immunosuppressants did not connect with a poorer prognosis of COVID-19.^[31]^ At the same time, more studies are needed to establish a causal relationship between them definitively.

The pancreatic damage produced by COVID-19 has received some attention. Potential mild pancreatic damage patterns were detected in COVID-19 patients in Wang F et al‘s study. These may be attributed to the direct viral involvement of the pancreas or secondary enzyme abnormalities.^[32]^ According to Liu F et al, SARS-CoV-2 may bind to ACE2 in the pancreas and cause pancreatic damage.^[33]^ Therefore, increased attention should be paid to the pancreas in patients with COVID-19, especially in severe cases. More studies should proceed to further definitively determine the correlation between COVID-19 and pancreatic injury.

Interestingly, among the100 top-cited papers, there were 2 studies on proton pump inhibitors (PPIs). Almario CV et al found that PPIs increase the risk of enteric infections.^[34]^ They suggested this may be related to PPI-induced hypochlorhydria. Later, Lee SW et al believed that patients taking PPIs had a higher risk of severe COVID-19 prognosis but were not more susceptible to SARS-CoV-2 than the general patient.^[35]^ More research into the relationship between COVID-19 and PPIs would be beneficial.

There are several limitations of this study. First, the citations of articles are influenced by a range of elements such as journals, the time span after publication, and authors’ influence. This might result in a missing of impactful research published recently. Second, we analyzed the data selectively. We mainly utilized the quantitative analysis approach. It is difficult to examine publications’ detailed contents and research design in bibliometric analysis. Thus, certain critical points and details may have been missed. Both of the considerations mentioned above may lead to biases in the results. Hence, the results should be interpreted with caution.

## 5. Conclusion

We conducted a bibliometric analysis of all eligible papers on COVID-19 and digestive disease in the present study. Liver, especially liver transplantation, and IBD were the 2 most influential research topics.

## Author contributions

**Conceptualization:** Xiaowei Tang.

**Data curation:** Shu Huang, Lei Shi.

**Formal analysis:** Shu Huang.

**Investigation:** Wensen Ren, Lei Shi.

**Project administration:** Muhan Lü.

**Resources:** Xinyi Zeng.

**Software:** Huifang Xia, Xinyi Zeng, Wensen Ren.

**Supervision:** Xian Zhou, Xiaowei Tang.

**Visualization:** Peiling Gan.

**Writing – original draft:** Peiling Gan.

**Writing – review & editing:** Peiling Gan, Xiao Pan, Xian Zhou.

## Supplementary Material
