# Supplementary material for: Global research trends in the COVID-19 and digestive disease: A review of visualization and bibliometric study
Source: Medicine (Baltimore). 2023 Jan 20;102(3):e32705. doi: 10.1097/MD.0000000000032705 (PMC9857270; doi:10.1097/MD.0000000000032705)
Supplement: Supplementary file 1 [file medi-102-e32705-s001.pdf]

Supplementary Table 1 List of the 100 top-cited articles in the COVID-19 and digestive disease.

| Rank | Articles | Number of citations (WoSCC) |
|---|---|---|
| 1 | Li Y, Xiao SY. Hepatic involvement in COVID-19 patients: Pathology, pathogenesis, and clinical implications. *J Med Virol*. 2020;92(9):1491-1494. | 878 |
| 2 | Jin X, Lian JS, Hu JH, et al. Epidemiological, clinical and virological characteristics of 74 cases of coronavirus-infected disease 2019 (COVID-19) with gastrointestinal symptoms. *Gut*. 2020;69(6):1002-1009. | 491 |
| 3 | Pan L, Mu M, Yang P, et al. Clinical Characteristics of COVID-19 Patients with Digestive Symptoms in Hubei, China: A Descriptive, Cross-Sectional, Multicenter Study. *Am J Gastroenterol*. 2020;115(5):766-773. | 441 |
| 4 | Tian Y, Rong L, Nian W, He Y. Review article: gastrointestinal features in COVID-19 and the possibility of faecal transmission. *Aliment Pharmacol Ther*. 2020;51(9):843-851. | 315 |
| 5 | Lin L, Jiang X, Zhang Z, et al. Gastrointestinal symptoms of 95 cases with SARS-CoV-2 infection. *Gut*. 2020;69(6):997-1001. | 307 |
| 6 | Fan Z, Chen L, Li J, et al. Clinical Features of COVID-19-Related Liver Functional Abnormality. *Clin Gastroenterol Hepatol*. 2020;18(7):1561-1566. | 291 |
| 7 | Zang R, Gomez Castro MF, McCune BT, et al. TMPRSS2 and TMPRSS4 promote SARS-CoV-2 infection of human small intestinal enterocytes. *Sci Immunol*. 2020;5(47):eabc3582. | 278 |

| | | |
|---|---|---|
| 8 | Wong SH, Lui RN, Sung JJ. Covid-19 and the digestive system. *J Gastroenterol Hepatol*. 2020;35(5):744-748. | 267 |
| 9 | Repici A, Maselli R, Colombo M, et al. Coronavirus (COVID-19) outbreak: what the department of endoscopy should know. *Gastrointest Endosc*. 2020;92(1):192-197. | 244 |
| 10 | Zuo T, Zhang F, Lui GCY, et al. Alterations in Gut Microbiota of Patients With COVID-19 During Time of Hospitalization. *Gastroenterology*. 2020;159(3):944-955.e8. | 229 |
| 11 | Boettler T, Newsome PN, Mondelli MU, et al. Care of patients with liver disease during the COVID-19 pandemic: EASL-ESCMID position paper. *JHEP Rep*. 2020;2(3):100113. | 222 |
| 12 | Boettler T, Marjot T, Newsome PN, et al. Impact of COVID-19 on the care of patients with liver disease: EASL-ESCMID position paper after 6 months of the pandemic. *JHEP Rep*. 2020;2(5):100169. | 222 |
| 13 | Han C, Duan C, Zhang S, et al. Digestive Symptoms in COVID-19 Patients with Mild Disease Severity: Clinical Presentation, Stool Viral RNA Testing, and Outcomes. *Am J Gastroenterol*. 2020;115(6):916-923. | 199 |
| 14 | Zhou J, Li C, Liu X, et al. Infection of bat and human intestinal organoids by SARS-CoV-2. *Nat Med*. 2020;26(7):1077-1083. | 178 |
| 15 | Chiu PWY, Ng SC, Inoue H, et al. Practice of endoscopy during COVID-19 pandemic: position statements of the Asian Pacific Society for Digestive Endoscopy (APSDE-COVID statements). *Gut*. 2020;69(6):991-996. | 167 |

| | | |
|---|---|---|
| 16 | Dhar D, Mohanty A. Gut microbiota and Covid-19- possible link and implications. *Virus Res*. 2020;285:198018. | 151 |
| 17 | Fix OK, Hameed B, Fontana RJ, et al. Clinical Best Practice Advice for Hepatology and Liver Transplant Providers During the COVID-19 Pandemic: AASLD Expert Panel Consensus Statement. *Hepatology*. 2020;72(1):287-304. | 138 |
| 18 | Bezzio C, Saibeni S, Variola A, et al. Outcomes of COVID-19 in 79 patients with IBD in Italy: an IG-IBD study. *Gut*. 2020;69(7):1213-1217. | 136 |
| 19 | Wang Y, Liu S, Liu H, et al. SARS-CoV-2 infection of the liver directly contributes to hepatic impairment in patients with COVID-19. *J Hepatol*. 2020;73(4):807-816. | 130 |
| 20 | Sultan S, Lim JK, Altayar O, et al. AGA Rapid Recommendations for Gastrointestinal Procedures During the COVID-19 Pandemic. *Gastroenterology*. 2020;159(2):739-758.e4. | 130 |
| 21 | Luo S, Zhang X, Xu H. Don't Overlook Digestive Symptoms in Patients With 2019 Novel Coronavirus Disease (COVID-19). *Clin Gastroenterol Hepatol*. 2020;18(7):1636-1637. | 128 |
| 22 | Mao R, Qiu Y, He JS, et al. Manifestations and prognosis of gastrointestinal and liver involvement in patients with COVID-19: a systematic review and meta-analysis [published correction appears in Lancet Gastroenterol Hepatol. 2020 Jul;5(7):e6]. *Lancet Gastroenterol Hepatol*. 2020;5(7):667-678. | 124 |
| 23 | Xie H, Zhao J, Lian N, Lin S, Xie Q, Zhuo H. Clinical characteristics of non-ICU hospitalized patients with coronavirus disease 2019 and liver injury: A | 121 |

| | | |
|---|---|---|
| | retrospective study. *Liver Int*. 2020;40(6):1321-1326. | |
| 24 | Gu S, Chen Y, Wu Z, et al. Alterations of the Gut Microbiota in Patients With Coronavirus Disease 2019 or H1N1 Influenza. *Clin Infect Dis*. 2020;71(10):2669-2678. | 116 |
| 25 | Sultan S, Altayar O, Siddique SM, et al. AGA Institute Rapid Review of the Gastrointestinal and Liver Manifestations of COVID-19, Meta-Analysis of International Data, and Recommendations for the Consultative Management of Patients with COVID-19. *Gastroenterology*. 2020;159(1):320-334.e27. | 109 |
| 26 | Kennedy NA, Jones GR, Lamb CA, et al. British Society of Gastroenterology guidance for management of inflammatory bowel disease during the COVID-19 pandemic. *Gut*. 2020;69(6):984-990. | 108 |
| 27 | Soetikno R, Teoh AYB, Kaltenbach T, et al. Considerations in performing endoscopy during the COVID-19 pandemic. *Gastrointest Endosc*. 2020;92(1):176-183. | 99 |
| 28 | Di Saverio S, Pata F, Gallo G, et al. Coronavirus pandemic and colorectal surgery: practical advice based on the Italian experience. *Colorectal Dis*. 2020;22(6):625-634. | 99 |
| 29 | Zhou Z, Zhao N, Shu Y, Han S, Chen B, Shu X. Effect of Gastrointestinal Symptoms in Patients With COVID-19. *Gastroenterology*. 2020;158(8):2294-2297. | 98 |
| 30 | Jothimani D, Venugopal R, Abedin MF, Kaliamoorthy I, Rela M. COVID-19 and the liver. *J Hepatol*. 2020;73(5):1231-1240. | 96 |

| | | |
|---|---|---|
| 31 | Neurath MF. COVID-19 and immunomodulation in IBD. *Gut*. 2020;69(7):1335-1342. | 94 |
| 32 | Hadi A, Werge M, Kristiansen KT, et al. Coronavirus Disease-19 (COVID-19) associated with severe acute pancreatitis: Case report on three family members. *Pancreatology*. 2020;20(4):665-667. | 93 |
| 33 | Iavarone M, D'Ambrosio R, Soria A, et al. High rates of 30-day mortality in patients with cirrhosis and COVID-19. *J Hepatol*. 2020;73(5):1063-1071. | 91 |
| 34 | Redd WD, Zhou JC, Hathorn KE, et al. Prevalence and Characteristics of Gastrointestinal Symptoms in Patients With Severe Acute Respiratory Syndrome Coronavirus 2 Infection in the United States: A Multicenter Cohort Study. *Gastroenterology*. 2020;159(2):765-767.e2. | 90 |
| 35 | Parasa S, Desai M, Thoguluva Chandrasekar V, et al. Prevalence of Gastrointestinal Symptoms and Fecal Viral Shedding in Patients With Coronavirus Disease 2019: A Systematic Review and Meta-analysis. *JAMA Netw Open*. 2020;3(6):e2011335. Published 2020 Jun 1. | 90 |
| 36 | Lui RN, Wong SH, Sánchez-Luna SA, et al. Overview of guidance for endoscopy during the coronavirus disease 2019 pandemic. *J Gastroenterol Hepatol*. 2020;35(5):749-759. | 86 |
| 37 | Monteleone G, Ardizzone S. Are Patients with Inflammatory Bowel Disease at Increased Risk for Covid-19 Infection?. *J Crohns Colitis*. 2020;14(9):1334-1336. | 82 |
| 38 | Rubin DT, Feuerstein JD, Wang AY, Cohen RD. AGA Clinical Practice Update on Management of Inflammatory Bowel Disease During the COVID-19 Pandemic: | 76 |

| | | |
|---|---|---|
| | Expert Commentary. *Gastroenterology*. 2020;159(1):350-357. | |
| 39 | Taxonera C, Sagastagoitia I, Alba C, Mañas N, Olivares D, Rey E. 2019 novel coronavirus disease (COVID-19) in patients with inflammatory bowel diseases. *Aliment Pharmacol Ther*. 2020;52(2):276-283. | 74 |
| 40 | Zhang H, Li HB, Lyu JR, et al. Specific ACE2 expression in small intestinal enterocytes may cause gastrointestinal symptoms and injury after 2019-nCoV infection. *Int J Infect Dis*. 2020;96:19-24. | 71 |
| 41 | Zuo T, Liu Q, Zhang F, et al. Depicting SARS-CoV-2 faecal viral activity in association with gut microbiota composition in patients with COVID-19. *Gut*. 2021;70(2):276-284. | 64 |
| 42 | Burgueño JF, Reich A, Hazime H, et al. Expression of SARS-CoV-2 Entry Molecules ACE2 and TMPRSS2 in the Gut of Patients With IBD. *Inflamm Bowel Dis*. 2020;26(6):797-808. | 63 |
| 43 | Mantovani A, Beatrice G, Dalbeni A. Coronavirus disease 2019 and prevalence of chronic liver disease: A meta-analysis. *Liver Int*. 2020;40(6):1316-1320. | 62 |
| 44 | Ferrey AJ, Choi G, Hanna RM, et al. A Case of Novel Coronavirus Disease 19 in a Chronic Hemodialysis Patient Presenting with Gastroenteritis and Developing Severe Pulmonary Disease. *Am J Nephrol*. 2020;51(5):337-342. | 62 |
| 45 | Bloom PP, Meyerowitz EA, Reinus Z, et al. Liver Biochemistries in Hospitalized Patients With COVID-19. *Hepatology*. 2021;73(3):890-900. | 61 |
| 46 | Colmenero J, Rodríguez-Perálvarez M, Salcedo M, et al. Epidemiological pattern, incidence, and outcomes of COVID-19 in liver transplant patients. *J Hepatol*. | 60 |

| | | |
|---|---|---|
| | 2021;74(1):148-155. | |
| 47 | Musa S. Hepatic and gastrointestinal involvement in coronavirus disease 2019 (COVID-19): What do we know till now?. *Arab J Gastroenterol*. 2020;21(1):3-8. | 59 |
| 48 | Huang JF, Zheng KI, George J, et al. Fatal outcome in a liver transplant recipient with COVID-19. *Am J Transplant*. 2020;20(7):1907-1910. | 58 |
| 49 | Gupta S, Parker J, Smits S, Underwood J, Dolwani S. Persistent viral shedding of SARS-CoV-2 in faeces - a rapid review. *Colorectal Dis*. 2020;22(6):611-620. | 57 |
| 50 | Tapper EB, Asrani SK. The COVID-19 pandemic will have a long-lasting impact on the quality of cirrhosis care. *J Hepatol*. 2020;73(2):441-445. | 55 |
| 51 | Snapiri O, Rosenberg Danziger C, Krause I, et al. Delayed diagnosis of paediatric appendicitis during the COVID-19 pandemic. *Acta Paediatr*. 2020;109(8):1672-1676. | 55 |
| 52 | Dolinger MT, Person H, Smith R, et al. Pediatric Crohn Disease and Multisystem Inflammatory Syndrome in Children (MIS-C) and COVID-19 Treated With Infliximab. *J Pediatr Gastroenterol Nutr*. 2020;71(2):153-155. | 54 |
| 53 | Yeoh YK, Zuo T, Lui GC, et al. Gut microbiota composition reflects disease severity and dysfunctional immune responses in patients with COVID-19. *Gut*. 2021;70(4):698-706. | 53 |
| 54 | Liu B, Wang Y, Zhao Y, Shi H, Zeng F, Chen Z. Successful treatment of severe COVID-19 pneumonia in a liver transplant recipient. *Am J Transplant*. 2020;20(7):1891-1895. | 53 |
| 55 | Alqahtani SA, Schattenberg JM. Liver injury in COVID-19: The current | 53 |

| | | |
|---|---|---|
| | evidence. *United European Gastroenterol J*. 2020;8(5):509-519. | |
| 56 | Lee IC, Huo TI, Huang YH. Gastrointestinal and liver manifestations in patients with COVID-19. *J Chin Med Assoc*. 2020;83(6):521-523. | 52 |
| 57 | Webb GJ, Marjot T, Cook JA, et al. Outcomes following SARS-CoV-2 infection in liver transplant recipients: an international registry study. *Lancet Gastroenterol Hepatol*. 2020;5(11):1008-1016. | 50 |
| 58 | Turner D, Huang Y, Martín-de-Carpi J, et al. Corona Virus Disease 2019 and Paediatric Inflammatory Bowel Diseases: Global Experience and Provisional Guidance (March 2020) from the Paediatric IBD Porto Group of European Society of Paediatric Gastroenterology, Hepatology, and Nutrition. *J Pediatr Gastroenterol Nutr*. 2020;70(6):727-733. | 49 |
| 59 | Sarin SK, Choudhury A, Lau GK, et al. Pre-existing liver disease is associated with poor outcome in patients with SARS CoV2 infection; The APCOLIS Study (APASL COVID-19 Liver Injury Spectrum Study). *Hepatol Int*. 2020;14(5):690-700. | 49 |
| 60 | Rutter MD, Brookes M, Lee TJ, Rogers P, Sharp L. Impact of the COVID-19 pandemic on UK endoscopic activity and cancer detection: a National Endoscopy Database Analysis. *Gut*. 2021;70(3):537-543. | 48 |
| 61 | Oyelade T, Alqahtani J, Canciani G. Prognosis of COVID-19 in Patients with Liver and Kidney Diseases: An Early Systematic Review and Meta-Analysis. *Trop Med Infect Dis*. 2020;5(2):80. Published 2020 May 15. | 48 |
| 62 | Marjot T, Moon AM, Cook JA, et al. Outcomes following SARS-CoV-2 infection | 47 |

| | | |
|---|---|---|
| | in patients with chronic liver disease: An international registry study. *J Hepatol*. 2021;74(3):567-577. | |
| 63 | Garrido I, Liberal R, Macedo G. Review article: COVID-19 and liver disease-what we know on 1st May 2020. *Aliment Pharmacol Ther*. 2020;52(2):267-275. | 47 |
| 64 | Du M, Cai G, Chen F, Christiani DC, Zhang Z, Wang M. Multiomics Evaluation of Gastrointestinal and Other Clinical Characteristics of COVID-19. *Gastroenterology*. 2020;158(8):2298-2301.e7. | 47 |
| 65 | Villapol S. Gastrointestinal symptoms associated with COVID-19: impact on the gut microbiome. *Transl Res*. 2020;226:57-69. | 44 |
| 66 | Becchetti C, Zambelli MF, Pasulo L, et al. COVID-19 in an international European liver transplant recipient cohort. *Gut*. 2020;69(10):1832-1840. | 44 |
| 67 | Müller JA, Groß R, Conzelmann C, et al. SARS-CoV-2 infects and replicates in cells of the human endocrine and exocrine pancreas. *Nat Metab*. 2021;3(2):149-165. | 43 |
| 68 | Cha MH, Regueiro M, Sandhu DS. Gastrointestinal and hepatic manifestations of COVID-19: A comprehensive review. *World J Gastroenterol*. 2020;26(19):2323-2332. | 43 |
| 69 | Kulkarni AV, Kumar P, Tevethia HV, et al. Systematic review with meta-analysis: liver manifestations and outcomes in COVID-19. *Aliment Pharmacol Ther*. 2020;52(4):584-599. | 42 |
| 70 | Fiorino G, Allocca M, Furfaro F, et al. Inflammatory Bowel Disease Care in the COVID-19 Pandemic Era: The Humanitas, Milan, Experience. *J Crohns Colitis*. | 42 |

| | | |
|---|---|---|
| | 2020;14(9):1330-1333. | |
| 71 | Zuo T, Zhan H, Zhang F, et al. Alterations in Fecal Fungal Microbiome of Patients With COVID-19 During Time of Hospitalization until Discharge. *Gastroenterology*. 2020;159(4):1302-1310.e5. | 41 |
| 72 | Zhao J, Liao X, Wang H, et al. Early Virus Clearance and Delayed Antibody Response in a Case of Coronavirus Disease 2019 (COVID-19) With a History of Coinfection With Human Immunodeficiency Virus Type 1 and Hepatitis C Virus. *Clin Infect Dis*. 2020;71(16):2233-2235. | 41 |
| 73 | Mönkemüller K, Fry L, Rickes S. COVID-19, coronavirus, SARS-CoV-2 and the small bowel. *Rev Esp Enferm Dig*. 2020;112(5):383-388. | 41 |
| 74 | Bajaj JS, Garcia-Tsao G, Biggins SW, et al. Comparison of mortality risk in patients with cirrhosis and COVID-19 compared with patients with cirrhosis alone and COVID-19 alone: multicentre matched cohort. *Gut*. 2021;70(3):531-536. | 41 |
| 75 | Almario CV, Chey WD, Spiegel BMR. Increased Risk of COVID-19 Among Users of Proton Pump Inhibitors. *Am J Gastroenterol*. 2020;115(10):1707-1715. | 41 |
| 76 | Li LY, Wu W, Chen S, et al. Digestive system involvement of novel coronavirus infection: Prevention and control infection from a gastroenterology perspective. *J Dig Dis*. 2020;21(4):199-204. | 40 |
| 77 | Lagana SM, De Michele S, Lee MJ, et al. COVID-19 Associated Hepatitis Complicating Recent Living Donor Liver Transplantation [published online ahead of print, 2020 Apr 17]. *Arch Pathol Lab Med*. 2020;10.5858/arpa.2020-0186-SA. | 40 |
| 78 | Ungaro RC, Brenner EJ, Gearry RB, et al. Effect of IBD medications on COVID- | 39 |

| | | |
|---|---|---|
| | 19 outcomes: results from an international registry. *Gut*. 2021;70(4):725-732. | |
| 79 | Kopel J, Perisetti A, Gajendran M, Boregowda U, Goyal H. Clinical Insights into the Gastrointestinal Manifestations of COVID-19. *Dig Dis Sci*. 2020;65(7):1932-1939. | 39 |
| 80 | Iacucci M, Cannatelli R, Labarile N, et al. Endoscopy in inflammatory bowel diseases during the COVID-19 pandemic and post-pandemic period [published correction appears in Lancet Gastroenterol Hepatol. 2021 Mar;6(3):e2]. *Lancet Gastroenterol Hepatol*. 2020;5(6):598-606. | 39 |
| 81 | Al-Ani AH, Prentice RE, Rentsch CA, et al. Review article: prevention, diagnosis and management of COVID-19 in the IBD patient. *Aliment Pharmacol Ther*. 2020;52(1):54-72. | 39 |
| 82 | Piano S, Dalbeni A, Vettore E, et al. Abnormal liver function tests predict transfer to intensive care unit and death in COVID-19. *Liver Int*. 2020;40(10):2394-2406. | 38 |
| 83 | Patel KP, Patel PA, Vunnam RR, et al. Gastrointestinal, hepatobiliary, and pancreatic manifestations of COVID-19. *J Clin Virol*. 2020;128:104386. | 38 |
| 84 | Park SK, Lee CW, Park DI, et al. Detection of SARS-CoV-2 in Fecal Samples From Patients With Asymptomatic and Mild COVID-19 in Korea. *Clin Gastroenterol Hepatol*. 2021;19(7):1387-1394.e2. | 38 |
| 85 | Tankel J, Keinan A, Blich O, et al. The Decreasing Incidence of Acute Appendicitis During COVID-19: A Retrospective Multi-centre Study. *World J Surg*. 2020;44(8):2458-2463. | 36 |
| 86 | Pawlak KM, Kral J, Khan R, et al. Impact of COVID-19 on endoscopy trainees: an | 35 |

| | | |
|---|---|---|
| | international survey. *Gastrointest Endosc*. 2020;92(4):925-935. | |
| 87 | Collard M, Lakkis Z, Loriau J, et al. Antibiotics alone as an alternative to appendectomy for uncomplicated acute appendicitis in adults: Changes in treatment modalities related to the COVID-19 health crisis. *J Visc Surg*. 2020;157(3S1):S33-S42. | 35 |
| 88 | Maggi U, De Carlis L, Yiu D, et al. The impact of the COVID-19 outbreak on liver transplantation programs in Northern Italy. *Am J Transplant*. 2020;20(7):1840-1848. | 34 |
| 89 | Occhipinti V, Pastorelli L. Challenges in the Care of IBD Patients During the CoViD-19 Pandemic: Report From a "Red Zone" Area in Northern Italy. *Inflamm Bowel Dis*. 2020;26(6):793-796. | 34 |
| 90 | Lagana SM, Kudose S, Iuga AC, et al. Hepatic pathology in patients dying of COVID-19: a series of 40 cases including clinical, histologic, and virologic data. *Mod Pathol*. 2020;33(11):2147-2155. | 33 |
| 91 | Hashemi N, Viveiros K, Redd WD, et al. Impact of chronic liver disease on outcomes of hospitalized patients with COVID-19: A multicentre United States experience. *Liver Int*. 2020;40(10):2515-2521. | 33 |
| 92 | Saigal S, Gupta S, Sudhindran S, et al. Liver transplantation and COVID-19 (Coronavirus) infection: guidelines of the liver transplant Society of India (LTSI). *Hepatol Int*. 2020;14(4):429-431. | 32 |
| 93 | Yip TC, Lui GC, Wong VW, et al. Liver injury is independently associated with adverse clinical outcomes in patients with COVID-19. *Gut*. 2021;70(4):733-742. | 31 |

| | | |
|---|---|---|
| 94 | Kotfis K, Skonieczna-Żydecka K. COVID-19: gastrointestinal symptoms and potential sources of SARS-CoV-2 transmission. *Anaesthesiol Intensive Ther*. 2020;52(2):171-172. | 31 |
| 95 | Huang W, Li C, Wang Z, et al. Decreased serum albumin level indicates poor prognosis of COVID-19 patients: hepatic injury analysis from 2,623 hospitalized cases. *Sci China Life Sci*. 2020;63(11):1678-1687. | 31 |
| 96 | Blach S, Kondili LA, Aghemo A, et al. Impact of COVID-19 on global HCV elimination efforts. *J Hepatol*. 2021;74(1):31-36. | 31 |
| 97 | Trottein F, Sokol H. Potential Causes and Consequences of Gastrointestinal Disorders during a SARS-CoV-2 Infection. *Cell Rep*. 2020;32(3):107915. | 30 |
| 98 | Ma C, Cong Y, Zhang H. COVID-19 and the Digestive System. *Am J Gastroenterol*. 2020;115(7):1003-1006. | 30 |
| 99 | Lee SW, Ha EK, Yeniova AÖ, et al. Severe clinical outcomes of COVID-19 associated with proton pump inhibitors: a nationwide cohort study with propensity score matching. *Gut*. 2021;70(1):76-84. | 30 |
| 100 | Gao F, Zheng KI, Wang XB, et al. Metabolic associated fatty liver disease increases coronavirus disease 2019 disease severity in nondiabetic patients. *J Gastroenterol Hepatol*. 2021;36(1):204-207. | 30 |
